# Treatments for Anxiety Disorders in Malaysia

**DOI:** 10.21315/mjms2019.26.3.2

**Published:** 2019-06-28

**Authors:** Jamilah Hanum Abdul Khaiyom, Firdaus Mukhtar, Oei Tian Po

**Affiliations:** 1 Department of Psychology, International Islamic University Malaysia, Gombak, Selangor, Malaysia; 2 Department of Psychiatry, Universiti Putra Malaysia, Serdang, Selangor, Malaysia; 3 School of Psychology and CBT Unit, Toowong Hospital, University of Queensland, Brisbane, St Lucia QLD, Australia; 4 Department of Psychology, James Cook University, 149 Sims Drive, Singapore

**Keywords:** anxiety, intervention, therapy, systematic review, Malaysia

## Abstract

This current study aims to systematically review the treatments for anxiety disorders in Malaysia. PsycINFO, MEDLINE databases, and 28 local journals were used to search published papers in this area. Eight articles were subjected to review after excluding 273 papers that did not meet the inclusion criteria. A total of 598 participants with various types of anxiety disorders were included in the review. Based on the findings, the combination of pharmacotherapy and psychotherapy provided better treatment outcomes if compared to psychotherapy or pharmacotherapy alone. The combination of selective serotonin reuptake inhibitors and cognitive behaviour therapy was considered as one of the most effective treatment to treat patients with anxiety disorders in Malaysia. This is in line with the clinical practice guidelines from the Ministry of Health Singapore and Canada. Even though there were some limitations in the methodology and reporting of the results, it can be concluded that efforts have been taken to conduct studies related to treatments for patients with anxiety disorders in Malaysia. Future studies are suggested to make conscious efforts to overcome these limitations.

## Introduction

One of the most commonly reported mental illness worldwide is anxiety disorder (AD) (1). As reported in a global meta-analytic review of 202 studies conducted in 94 countries (1–2), approximately one-eighth to one-sixth of the world’s population will experience AD in their lifetime. Meanwhile, according to Baxter et al. (3), AD is the sixth leading cause of disability, in low-, middle- and high-income countries. The burden of AD has also been reported through the increase in the expenses of health care services and the economic cost for AD since 2005 (3); in 2010, the economic cost for AD was 74.4 billion Euros per year in Europe (4), and in Malaysia anxiety is one of the most commonly reported and growing mental health problems (5). According to the Fourth National Health Morbidity Survey (NHMS-IV), the prevalence rate of generalised anxiety disorder (GAD) was 1.7%, which is comparable with international figures (1.9%–2.5%) (5). Based on the systematic review by Abdul Khaiyom, the prevalence rates of anxiety among students, general and clinical populations in Malaysia range between 1% to 67.6%, (unpublished data). This indicates the paramount need to ensure that patients with AD receive proper treatments.

There are several treatments available for people with AD. These treatments can be categorised as pharmacological treatment, psychological treatment, or a combination of both. The selection between these types of treatments depends on factors such as i) patient’s preference and motivation, ii) treatment options availability, iii) adverse medications effects, iv) ability/availability of patients to engage/commit in the treatment, v) severity of illness, the presence of co-morbid medical or psychiatric disorders, vi) financial considerations, and vii) clinicians’ expertise (6–7).

### Pharmacological Treatments for Anxiety Disorders

A systematic review of 510 randomised controlled trials (RCT) by the World Federation of Biological Psychiatry (WFSBP) reported that selective serotonin re-uptake inhibitors (SSRIs) is one of the first-line pharmacological treatments for AD [i.e., panic disorder (PD), agoraphobia (A), GAD, specific phobia (SP), and social anxiety disorder (SAD)] (7). Serotonin-norepinephrine reuptake inhibitors (SNRIs), such as Venlafaxine is also considered the first-line treatment for AD (6–8) due to the full evidence obtained from RCT. Besides that, they also have a good risk-benefit ratio (7). Furthermore, Bandelow et al. (9) found large treatment effect size (*d* = 2.09–2.25) in the meta-analytic review of 85 studies that used SSRIs or SNRIs as a treatment for AD. These drug treatments are also being practised and outlined in the Singapore Ministry of Health (MOH) Clinical Practice Guideline (CPG) for AD (8), Health Canada CPG for AD (6), and also British Association for Psychopharmacology guidelines for AD (10). On the other hand, the WFSBP and Singapore MOH CPG do not recognise the use of psychopharmacological drugs as a standard treatment in simple SP cases (7–8), but SSRIs can be tried in severe cases (7).

Apart from the above drugs, tricyclic anti-depressants (TCAs), benzodiazepines (BZD) (7, 10), MAO inhibitors (MAOIs) (10), and noradrenergic and specific serotoninergic antidepressant (NasSA) (6–7) are also being used in the second-line pharmacological treatment of AD due to their moderate risk-benefit ratio (7). In the meta-analytic review of 57 studies, Bandelow et al. (9) reported large treatment effect size (*d* = 1.83–2.15) for both TCA and BZD, while based on the results of the systematic review by WFSBP and Singapore MOH CPG, the efficacy of TCAs, mainly for imipramine and clomipramine, has been well proven in the treatment of PD and GAD (7–8). Similar to TCAs, BZD has also been used in the management of PD and GAD (7) while the use Phenelzine, a form of MAOIs, is evident in the treatment of SAD and PD (7), (10). Hydroxyzine, a drug under NasSA is also being used in GAD management due to its sedating effects (6–8).

### Psychological Treatments for Anxiety Disorders

There is strong evidence to support the use of psychotherapy as a first-line treatment for AD. Canadian Psychological Association in their meta-analyses on psychological treatments for AD argued that the strength of psychotherapeutic effects for AD is similar or superior to what usually found in the pharmacotherapy effect (11). Moreover, as poor compliance from patients could result in adverse effects associated with pharmacological treatments, Hunsley et al. (11) reported that many CPGs have considered psychological treatments as the first-line treatment for AD. However, this is only relevant if the psychological treatments have been thoroughly evaluated, made available, preferred by the patient, and being conducted by suitably trained and supervised therapists (6).

There are many types of psychological treatments for AD including Cognitive Behaviour Therapy (CBT) and Relaxation Training (RT). In the meta-analytic review by Hoffmann et al. (12), CBT has been found to be the most researched psychological treatment and is endorsed as the current gold standard of psychotherapy (13). Similar to the above finding, a meta-analytic review of 108 trials of CBT for AD concluded that CBT effects have been efficacious across AD, and greater in active control treatments than non-treatments (14). Therefore, it is not surprising to find that CBT is considered as the first-line psychological treatment in many guidelines (10), (15–16).

In the meantime, many previous studies on psychological treatment of AD have focused on RT. There are many forms of RT for AD treatment, such as (i) deep breathing (17), (ii) progressive muscular relaxation, (iii) autogenic (18), and (iv) guided imagery (19). A considerable amount of literature has confirmed the effectiveness of RT in the treatment of GAD (20–22), PD (23), SAD (24–25), and SP (e.g., fear of flying and dentist phobia) (26–27). Additionally, two meta-analytic reviews have supported the effectiveness of RT as a treatment for AD in both individual and group sessions with effect sizes between moderate (*d* = 0.57) (28) and large (*d* = 1.36) (9).

### Combination of Psychological and Pharmacological Treatments for Anxiety Disorder

As pharmacological and psychological treatments have their own accessibility and effectiveness, the combination of both (termed as psychopharmacological treatment) has been shown to be a clinically desired AD intervention strategy (7). Its effectiveness was proven in the meta-analytic review of 16 studies using CBT and medication and large effect size (*d* = 2.12) was found (9).

It is also imperative to note that psychopharmacological treatment for AD has shown short-term and long-term effectiveness with lower drop-out rate, even after the termination of pharmacotherapy (9), (29–30) and psychopharmacological treatment for AD is also more cost-effective (31–32). Meanwhile, despite the reported advantages of combined treatment, some studies reported that combined treatment has no advantage that some of the benefits of combined treatment are accounted for by pharmacotherapy alone or psychotherapy alone (33).

Nevertheless, it is important to remember that the investigations on the combination of treatments for AD are still relatively novel where much of the cited evidence is from the combination of CBT and medications on PD, rather than RT and medications. Therefore, this current study aims to systematically review all available treatments for AD in Malaysia with the hopes of bringing additional information and potentially new ways of combining these treatments to change the now-cautious recommendations.

## Methods

### Literature Search

In June 2016, PsycINFO and MEDLINE databases were searched using the search terms *anxiety, panic, phobia, distress*, and were combined with the terms treat*, interven*, therapy and *Malaysia* as identifiers. The scope of the search was limited to the title of scientific articles published in English or Malay from 1980 to 2015, with no restriction of the subject area. In addition, 28 local journals were manually searched and scrutinised between June to December 2016, these journals are Malaysian Journal of Psychiatry, International Journal of Public Health Research, Jurnal Sains Kesihatan Malaysia, Jurnal Psikologi Malaysia, Malaysian Journal of Medicine and Health Sciences, International Medical Journal of Malaysia, and The Malaysian Journal of Medical Sciences.

### Inclusion Criteria

The studies included are those which (i) contained treatments used to treat patients with AD, (ii) provided information on the outcomes of the treatment (iii) conducted for Malaysian population, (iv) conducted in Malaysia, and (v) published in a peer-reviewed journal.

### Data Extraction

Data were extracted independently by one reviewer and entered into data extraction forms designed for the review. Another reviewer was consulted regarding any discrepancies which were resolved through discussion until a consensus was reached.

### Encoding Results

The included studies were sorted based on the order of publication year. The details of the studies were extracted into ten features: (i) author/s and year of publication, (ii) participants characteristics and settings, (iii) measures used, (iv) assessment time, (v) design of the study, (vi) intervention/s used, (vii) clinicians who conducted intervention, (viii) attrition rate, (ix) analysis involved, and (x) findings.

The encoding criteria were used to understand the trend of research design in investigating the efficacy or effectiveness of treatments for AD in Malaysia. The results will be analysed and utilised as the basis for future improvements for both clinical and research purposes.

## Results

### Search Results

**Figure 1:**
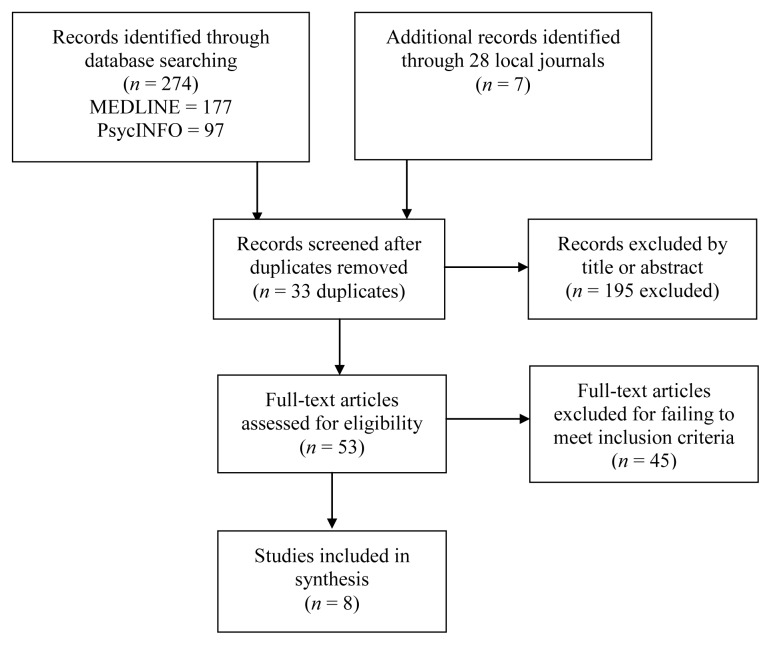
Study selection flowchart

Out of the 281 articles found, 273 were excluded for not meeting the inclusion criteria. Therefore, eight articles were subjected to the review (see Figure 1 for the articles selection flowchart).

### Description of Studies

Table 1 shows the included studies on the treatments used for patients with AD in Malaysia. The earliest study on the treatment outcome for AD was in 1992 and the latest in 2007. No other studies were found between 2007 and until the literature search was conducted. Four out of eight studies were conducted by the same person or team (i.e., Azhar and colleagues) (34, 37, 39–40).

### Study Design

Four of the studies (Study 1, 4, 5, and 7) (34, 37–38, 40) reported the use of RCT design for conducting trials. Meanwhile one study (Study 6) (39) was conducted using non-RCT design and another study (Study 3) (36) is a controlled trial but the random assignment of participants was not reported. Furthermore, two studies (Study 2 and 8) (35), (41) are case studies.

### Participants Involved

A total of 598 patients with AD were included in the review, with sample size ranging from 1 to 200. Most of the studies involved patients with a specific diagnosis of AD, such as PD and GAD. However, only one study (Study 6) (39) combined patients with heterogeneous AD (i.e., GAD, SAD, and Panic). Moreover, two of the studies (Study 5 and 6) (38–39) include patients with depression as participants.

In terms of the study setting, six out of eight studies were conducted in Kelantan (a state in the east coast of Malaysia) and almost all the studies were conducted in a university-based hospitals or clinics. The age range of patients with AD was between 24 and 40 years old. However, the results are not definitive since some studies did not report the data. Moreover, there is limited information on gender and ethnicities of the patients.

### Measures Used

A majority of studies reported the use of Diagnostic and Statistical Manual of Mental Disorders (DSM) as a diagnostic measure to assess AD. However, only Study 3 (36) reported the use of a Structured Clinical Interview for DSM Disorders in confirming the diagnosis of patients with AD.

In the meantime, most of these studies used more than one self-report measure and most used were anxiety symptoms measure such as Hamilton Anxiety Rating Scale (HARS) and Beck Anxiety Inventory (BAI). Some of the studies have incorporated the use of depressive symptoms measure to assess the comorbid symptoms of depression among patients with AD. The most used measures to assess depressive symptoms are Hamilton Depression Rating Scale (HDRS) and Beck Depression Inventory (BDI).

Apart from the above measures, other ecologically valid measures relevant to anxiety were minimally used in the studies. For example, only one study used WHO Quality of Life-BREF measure (WHOQOL-BREF), two studies reported the use of cognitive-based measures (i.e., Catastrophic Belief Score and Daily Schedule of Automatic Thoughts Record Form), and two studies used validated religiosity scale in the Malay language.

### Time of Assessments

A majority of these studies conducted baseline assessment to assess the patients’ conditions prior to the treatment; middle-treatment assessments were conducted by Study 2 (35) and Study 4 (37) to measure if there were treatment gains prior to the completion of the treatment received. Three studies specified the conduct of post-treatment assessments (i.e., immediately after the treatment was completed) to measure the effectiveness of treatment received by patients.

Some of the studies also specified the time of assessments (e.g., at 4th week, at 12th week, at 26th week, at 3-month, and at 6-month). However, due to unclear reporting on the intervention status (i.e., patients are still receiving the intervention or not), it could not be confirmed if the assessments are post-treatment assessments. Only study 8 (41) reported a confirmed follow-up assessments using phone calls and letters on the 6th and 12th month after the completion of treatment.

### Details of the Interventions Used

Interventions used to treat AD in Malaysia can be categorized in three general approaches: (i) pharmacotherapy alone, (ii) psychotherapy alone, and (iii) combination of pharmacotherapy and psychotherapy.

#### i. Pharmacotherapy alone

One of the treatment arms in Study 4 (37) used only fluvoxamine (FVX) to treat patients with AD. The dosage of FVX was specified (i.e., start at 50mg/day to 200mg/day) and the treatment session was reported (i.e., nine sessions).

#### ii. Psychotherapy alone

Four studies only used psychotherapy as either a part of their treatment arm, or as the only treatment option. For example, Study 2 (35), Study 4 (37), and Study 6 (39) used CBT as their treatment. On the other hand, Study 8 (41) used hypnosis and RT.

A majority of the studies reported the number of treatment sessions, for example, Study 2 (35) and Study 4 (37) conducted twelve and nine CBT sessions, respectively. Whereas, Study 8 (41) conducted only one session of hypnosis and RT. None of the studies informed the duration of each treatment session.

Only Study 2 (35) detailed out the treatment protocol used while Study 4 (37) only reported the guidelines. However, none of the studies reported the use of specific published treatment manual as the reference for their treatment protocol.

#### iii. Combination of pharmacotherapy and psychotherapy

Five studies used the combination of pharmacotherapy and psychotherapy. Study 1 (34) and Study 4 (37) used CBT as the psychotherapy and the pharmacotherapy used were from different types of SSRIs (i.e., escitalopram-ESC, sertraline-STR, fluoxetine-FXT, and FVX). On the other hand, Study 3 (36), Study 5 (38) and Study 7 (40) used religious and cultural-based supportive psychotherapy. Study 3 also reported the incorporation of Beck’s Cognitive Model as part of the elements in the therapy. Meanwhile, in these studies BZD was used as pharmacotherapy treatment. From these five studies, only Study 1 (34) and Study 4 (37) reported the dosage of pharmacotherapy given.

The duration of pharmacotherapy ranged between seven to 16 weeks, whereas, the psychotherapy session ranged between nine to 16 sessions (37), (40). However, the information is not definitive since Study 3 (36) and Study 5 (38) did not report the number of their treatment sessions while only Study 7 (40) reported the duration of each treatment session, (i.e., 45 min).

All five studies generally described the elements used in the therapy. However, there is no report on the use of specific published treatment manual as the reference for their treatment protocol and the studies did not report whether the psychotherapy were conducted in individual or group format.

### Clinician

Almost all of the pharmacotherapy and psychotherapy treatments in the studies were conducted by psychiatrists, except for Study 2 (35). In the study, the treatment was conducted by a clinical psychologist. Meanwhile, Study 7 (40) did not report the involvement of any clinician in the treatment. None of the studies reported how many clinicians involved in conducting the treatments.

### Attrition

All studies, except for Study 1 (34), reported the attrition rate between 0% and 50%. In general, patients’ drop-out occurred due to the patients’ inability to complete the minimum requirement of the therapy (e.g., six months of therapy). Moreover, only Study 4 (37) mentioned the time relative to the attrition rate (i.e., during the treatment, after the completion of treatment, during follow-up).

### Analysis

All of the studies, except Study 2 (35) and Study 8 (41) which utilised case study design, conducted significant testing to analyse the effectiveness of treatments received by the patients. Out of four studies utilising RCT design, only Study 4 conducted intention-to-treat (ITT) analysis while other two studies, Study 5 (38) and Study 7 (40), used per-protocol (PP) analysis and one study (Study 1) did not report the use of either ITT or PP analysis (34).

None of the studies reported the effect size of the treatment outcome. However, the readers may manually calculate the effect size using the mean, standard deviation, and number of samples in the group provided.

### General Findings

All studies reported positive improvements in patients with AD after receiving the treatments, but only Study 4 (37) able to compare the effectiveness of the three treatment approaches (i.e., a combination of pharmacotherapy and psychotherapy, psychotherapy alone, and pharmacotherapy alone) and it was found that the combination of CBT with FVX, or CBT alone provide greater improvements to patients with AD if compared to FVX alone.

Furthermore, Study 6 (39) found that CBT is more effective if compared to supportive psychotherapy and patients were found to improve continuously at the 6-month follow-up. Other than the above, RSCP or RSP with a combination of BZD (refer Study 3, Study 5, and Study 7) (36, 38, 40) are more effective if compared to the treatment using supportive psychotherapy with BZD. This provides evidence on the importance of religion and socio-cultural elements as part of the treatment component for patients with AD in Malaysia.

## Discussion

This study aims to systematically review the treatments for anxiety disorders in Malaysia. Based on the review, in Malaysia, AD is treated using either pharmacological treatment, or psychological treatment, or the combination of both. Significant improvement with large effect sizes were seen in the patients with PD treated using combination of psychotherapy and pharmacotherapy (CBT with FVX) or psychotherapy alone (CBT) (37). This is in line with the study conducted by Bandelow et al. (7) who found the combination between CBT and medication was shown to have the best treatment outcome. In addition, Katzman et al. (6) in their clinical practice guidelines for the management of anxiety argued that pharmacological treatments can enhance the efficacy of CBT. Nonetheless, the knowledge about the use of combination treatments for AD relies heavily upon empirical studies on PD using CBT and medications.

In addition, this review revealed that either RSCP or RSP with a combination of SP and pharmacotherapy were more effective (refer to Study 3, 5, and 7) than using SP with pharmacotherapy only (36, 38, 40). This illustrates that religio-socio-cultural aspects play a vital role in the treatment of AD in Malaysia since Malaysians are deemed as a cultural and religion oriented nation (36). Furthermore, Razali et al. (36) suggested that therapeutic relationships between patient and mental health service providers can be strengthened when cultural aspect is taken into considerations. Thus, incorporating RSCP or RSP in the treatment of AD may have great benefit, since it has been long known that cultural background may influence the therapeutic relationships between patients and healthcare professionals (36).

### Limitations and Recommendations for Improvements

There are several limitations found on the studies reviewed. It is hoped that future studies will be able to bridge the gaps found in the literature.

### Time of studies

It was surprising to find that the most recent research on treatments of AD in Malaysia was published in 2007. Moreover, most published research found have been conducted primarily by the similar team of researchers (i.e., Azhar–a psychiatrist-and his colleagues). Hence, this area of research is still in an infantile stage and there are vast opportunities for future discoveries. Based on the high prevalence rates of anxiety in Malaysia (5) (unpublished data), future research needs to focus on treatment outcome studies. This is to ensure health care providers are ready to provide empirical and evidence-based treatments for patients with AD in Malaysia.

### Power and sample size calculation

Most of the studies reviewed have a very small sample size and this is considered as a serious limitation. Besides that, none of the studies provided power and sample size calculations in their paper, hence, the results may be biased to Type-I error whereby researcher may conclude that the supposed treatment effectiveness exists when in fact it does not (42–43).

### Location of studies

Most of the studies were conducted in the eastern part of Malaysia and there are very few studies conducted in the central part of Malaysia, especially in the Klang Valley which is the most populated area in Malaysia besides constituting the largest urban centre in the country (44). According to Institute of Public Health (5), mental health problems, including AD were found to be more prevalent in urban areas compared to rural areas. Therefore, it is suggested that future research on treatment outcome is conducted in the central part of Malaysia, especially in the Klang Valley.

Since most of the studies were conducted in university-based hospitals or clinics (i.e., academic or research setting), it is not certain that the treatments used in the studies could be generalised and transported to public health settings, such as government hospitals. Future research is encouraged to bridge this gap by conducting research on different settings to test the generalisations of treatments and its results.

### Details of participants

Most of the studies reviewed did not report the information related to gender and ethnicities. Therefore, the important demographic information of the patients were missing, this has limit our understanding on the epidemiology of patients with AD in Malaysia.

### Assessments used and issue of multiplicity adjustments

While some of the studies used more than one anxiety symptoms measures, none of the studies reported their primary outcome measure and the adjustment for multiple comparisons (e.g., using Bonferroni-type adjustment). According to Tyler et al. (45), no correction for a multiplicity of treatment outcomes may inflate the risk of Type-I error.

Meanwhile, a small number of studies used the depressive symptoms measure to assess the effect of treatments on the comorbidity symptoms and very few studies used other ecologically valid measures such as quality of life and cognitive measures. Convergence in outcomes across these numerous measures will provide more convincing evidence of positive treatment outcomes. Therefore, there is a need to evaluate the effectiveness of treatments for AD in Malaysia with a broader range of outcome measures.

Moreover, none of the reviewed studies reported the use of validated outcome measures for anxiety symptoms, depressive symptoms, and quality of life. Based on the limitations presented, future studies are encouraged to be more careful in the utilisation of outcome measures to ensure the validity of the results.

### Reporting of assessment time, study design, and treatment procedures

The reporting of most studies related to assessment time (i.e., baseline, mid, post, and follow-up treatment) was not clear which could impose difficulties for future researchers to design related studies.

Furthermore, even though a majority of the studies claimed that they used RCT as their study design, the studies did not specifically report the procedures of enrollment, allocation and randomisation, follow-up, and analysis. For example, majority of the studies lacked in terms of (a) reporting of the treatment procedures, such as the duration of each treatment session, (b) reporting on the use of treatment manual to confirm standardisation of treatment received by each patients, (c) information on how many clinicians involved in conducting the treatments, (d) information whether the treatment being conducted in individual or group format and (e) information related to assessment time and attrition rate. This raised concern about the quality of the RCT studies being conducted. Therefore, future studies are very much encouraged to conduct and report RCT studies in accordance with CONSORT guideline (46).

### Clinicians involved in the studies

The majority of the studies were conducted by psychiatrists who were known to be trained in the prescription of medications as part of mental disorders management. According to Ng (47), conducting psychological assessments and psychological interventions are not part of the compulsory training received by the psychiatrist. Therefore, clinical psychologists who are trained in both psychological assessments and psychological interventions such as CBT and RT are very much encouraged to collaborate with psychiatrists in conducting treatment outcome studies related to AD. This is supported by Abdul Wahab Khan (48) who argued that clinical psychologists are highly needed in Malaysia due to their ability to both diagnose and provide psychological interventions for mentally ill patients.

### Analysis used in the studies

The current systematic review found that limited RCT studies used ITT analysis. The issues of non-compliance protocol deviations, attrition or withdrawal, or anything that happens after randomisation treatment assignment are balanced by the use of ITT analysis (49). Therefore, RCT studies need to use ITT as part of their statistical analysis.

Moreover, none of the studies reported treatment effect sizes which are the key product of a research investigation. Effect size is the magnitude of the difference between treatment groups (50–51). Even though a significant statistical value (e.g., P < 0.05) can inform the reader whether an effect exists, it will not reveal the size of the effect. Moreover, it is vital to report the effect size for treatment outcome studies in Malaysia since it will assist future researchers to calculate the number of participants sufficient in their studies. This is to avoid a Type-II error (the probability of concluding there is no effect when one actually exists).

In addition, only one study provides clinical significant change analysis (CC) and none of the studies provided reliable change analysis (RC). These two analyses are important for researchers and clinicians to communicate with patients on the treatment effects pragmatically. Due to this, future research is encouraged to include not only significant testing and effect sizes in their analysis but also RC and CC.

## Conclusion

Even though the studies reviewed have shown some limitations in the methodology and reporting of the results, it can be concluded that these studies have taken the efforts to conduct studies on treatments for patients with AD in Malaysia. Based on the review, the combination of pharmacotherapy and psychotherapy provided better treatment outcome if compared to psychotherapy or pharmacotherapy alone. Furthermore, it can be seen that CBT treatment was considered as one of the treatments of choice for psychological intervention to treat patients with AD in Malaysia. This is in-line with the clinical practice guidelines from Singapore (8) and Canada (6). However, it is recommended for future studies to make conscious efforts to minimise the weaknesses that have been discussed previously.

## Figures and Tables

**Table 1:** Characteristics of included studies

Study	Participants	Measures	Time	Design	Interventions	Clinician	Attrition	Analysis	Findings
Study 1 (34)	DSM-IV PD Specialist hospital, KL, *N* =95 *M* Age = 32 Gender NR Ethnicity NR	*Diagnostic* DSM-IV *Self-Reports* HARS BAI Cbs Pf/week WHOQOL-BREF	T1 12-wk	RCT CBT+ESC: 33 CBT+STR: 31 CBT+FXT: 31	CBT+ESC: ESC start at 10 mg/day–20 mg/day (if x side effects) CBT+STR: STR start at 50 mg/day–100 mg/day (if x side effect) CBT+FXT: FXT start at 20 mg/day–40 mg/day (if no side effect) All patients were seen weekly and received CBT.	Psychiatrist	NR	Sig. test	+ve CBT+ESC > +ve +++
Study 2 (35)	PDA University health psychology clinic, KL, *N =* 1 Age = 24 Male Ethnicity NR	*Diagnostic* NR *Self-Reports* DRAT BAI BDI AS-MMPI2S STAI	T1 T2(6th session) T3	Case study	CBT 12 sessions Provide descriptions of a treatment protocol for the therapy.	Clinical psychologist	Nil	Pre-Mid-Post (comparisons using raw data and clinical classification)	+ve Improved from severe anxiety at Ti to minimal anxiety at T3
Study 3 (36)	DSM-III-R GAD University psychiatric clinic, Kelantan, *N =* 200 Muslims *M* age = NR Gender NR Ethnicity = Malay	*Diagnostic* SCID for DSM-III-R *Self-Reports* HARS	T1 4-wk 12-wk 26-wk	Controlled trial No report on random allocation BZD+SP+RLX+RCPwCM: 50 BZD+SP+RLX: 50 Each group has equivalent of religious and non-religious patients.	Provided brief info on the use of BZD and SP. Provided brief guideline on the use RCPwCM in the study group. All patients were followed up for six months; and reviewed weekly (in the 1st 4-wk), fortnightly (until 12-wk), and then monthly.	Psychiatrist	Total: 17-5%	Sig. test	+ve BZD+SP+RLX+RCPwCM > +ve at 4-wk & 12-wk No sig. diff. at the end of 26-wk for both of the group and type of patients (i.e., religious versus non-religious)
Study 4 (37)	DSM-IV PD University hospital, Kelantan, *N* = 66 *M* age = 32 Gender NR Ethnicity NR	*Diagnostic* DSM-IV *Self-Reports* BAI BDI HARS HDRS Pf/week Cbs *Others* Bp, Pr	T1 every session	RCT CBT+FVX: 22 CBT: 22 FVX: 22	All treatment groups, CBT+FVX, CBT alone, & FVX alone: 9 sessions, CBT: Guidelines for therapy were from other studies. FVX: Start at 50 mg/day– 200 mg/day (if x side effects)	Psychiatrist	Total: 22.7% (during follow-up)	ITT Sig. test	+ve CBT+FVX & CBT alone > +ve +++
Study 5 (38)	DSM-III-R GAD & MDD(x psychosis) University hospital, Kelantan, *N* = 240 (GAD *n* = 120) *M* age NR Gender NR Ethnicity: 100% Malay	*Diagnostic* DSM-III-R *Self-Reports* HARS HDRS Religious questionnaire (Malay valid.)	T1 4-wk 12-wk 26-wk	RCT RSCP+SP+RLX+BZD for GAD: 54 SP+RLX+BZD for GAD: 49	Both treatment groups, RSCP+SP+RLX+BZ D & SP+RLX+BZD for GAD: Patients were followed for 6-mth. RSCP&SP: Provide descriptions of the elements used in the therapy. BZD: ≤ 6 wk.	Psychiatrist	For GAD: 14%	PP Sig. test	+ve continuously improved at 6-mth RSCP+SP+BZD > +ve at 1-,3-,& 6-mth +++
Study 6 (39)	DSM-IV Depression (x psychosis), GAD, SAD, Panic, OCD University hospital, Kelantan, *N*=116 (GAD *n* = 38), *M* Age = 37 (for GAD), Gender: 53.8% female (for GAD), Ethnicity NR	*Diagnostic* DSM-IV *Self-Reports* HARS HDRS	2-wk after treatment, 3-mth 6-mth	NRCT For GAD; CBT: 26 SP: 12 CBT: SAD: 4 Panic: 8 OCD: 2	For GAD: No report on both CBT and SP procedures and durations of therapy.	Psychiatrist	GAD: 7.7% SAD: 50% Panic: 0% OCD: 50%	Sig. test	+ve continuously improved at 6-mth CBT > +ve at 3-mth & 6-mth
Study 7 (40)	DSM-III-R GAD University hospital, Kelantan, *N* = 77 *M* age = 40 Gender NR Ethnicity NR (all are Muslims)	*Diagnostic* DSM-III-R *Self-Reports* Religious questionnaire HARS	T1 3-mth 6-mth	RCT RSP+BZD:31 SP+BZD:31	Both treatment groups, RSP+BZD &SP+BZD: 12–16wk sessions, 45 min/wk RSP & SP: Provide descriptions of the elements used in the therapy. BZD: ≤ 8 wk.	Psychiatrist	Total: 19.5%	PP Sig. test	+ve continuously improved at 6-mth RSP+BZD > +ve at 3-mth +++
Study 8 (41)	Insomnia & Anxiety University hospital, Kelantan, *N*=1 Age = 29 Female Ethnicity NR	NR	T3 T4 (6-mth & 12-mth)	Case study	Hypnosis+RLX+Stimulus control+correction of misconception, 1 session	NR	Nil	Qualitative descriptions	+ve continuously improved at 12-mth

Notes: NR = Not reported.

Participants: DSM-IV = Diagnostic and Statistical Manual of Mental Disorders 4th ed.; DSM-III-R = DSM 3rd ed-Revision; PD = Panic disorder; GAD = Generalised anxiety disorder; PDA = Panic disorder with Agoraphobia; SAD= Social anxiety disorder; OCD = Obsessive-compulsive disorder; KL = Kuala Lumpur.

Measures: SCID = Structured clinical interview; BAI = Beck anxiety inventory; BDI = Beck depression inventory; HARS = Hamilton Anxiety Rating Scale; HDRS = Hamilton Depression Rating Scale; PF = Panic frequency per week; Cbs = Catastrophic belief score; Bp = Blood pressure; Pr = Pulse rate; DRAT = Daily schedule of automatic thoughts records form; AS-MMPI2s = Anxiety Scale of the Minnesota Multiphasic Personality Inventory-2 Supplementary Scale; STAI = State-trait anxiety inventory; Malay valid. = the scale has been validated in Malay language version; WHOQOL-BREF = World Health Organization Quality of Life-Brief version.

Time = Assessment Time: T1 = Baseline; T2 = Mid-treatment assessment; T3 = Post-treatment assessment; T4 = Follow-up assessment wk = week; mth = month.

Design &/ Intervention: RCT = Randomised controlled trial; NRCT = Non-randomised controlled trial. Interventions: CBT = Cognitive behaviour therapy; RCPwCM = Religious-Cultural Psychotherapy with Beck’s Cognitive Model; RSP = Religious & Supportive Psychotherapy; SP = Supportive Psychotherapy; RSCP = Religious-Sociocultural Psychotherapy; RLX = Relaxation Exercise; ESC = Escitalopram; STR = Sertraline; FXT = Fluoxetine; FVX = Fluvoxamine; BZD = Benzodiazepines; wk = week, min = minutes.

Analysis: ITT = Intention-to-treat analysis; PP = Per-protocol analysis; Sig. test = Significant testing for mean difference (e.g., *t*-test, ANOVA); CC = Report clinical significance change.

Findings: +ve = interventions used are generally effective in improving the patients’ conditions during assessments/post-treatment/ at follow-up; Sig. diff. = significant difference/s; +++ = Experimental group has large treatment effect size at post-treatment.
